# Heterozygous Deletion of the *SHOX* Gene Enhancer in two Females With Clinical Heterogeneity Associating With Skewed XCI and Escaping XCI

**DOI:** 10.3389/fgene.2019.01086

**Published:** 2019-11-06

**Authors:** Yixi Sun, Yuqin Luo, Yeqing Qian, Min Chen, Liya Wang, Hongge Li, Yu Zou, Minyue Dong

**Affiliations:** ^1^Department of Reproductive Genetics, Women’s Hospital, School of Medicine, Zhejiang University, Hangzhou, China; ^2^Key Laboratory of Reproductive Genetics, Ministry of Education, Zhejiang University, Hangzhou, China; ^3^Key Laboratory of Women’s Reproductive Health of Zhejiang Province, Zhejiang University, Hangzhou, China; ^4^Department of Diagnostic Radiology, Women’s Hospital, School of Medicine, Zhejiang University, Hangzhou, China

**Keywords:** *SHOX* gene enhancer, Leri-Weill dyschondrosteosis, skewed X-chromosome inactivation (XCI), clinical heterogeneity, *HUMARA* assay, escaping X-chromosome inactivation (XCI)

## Abstract

Skewed X-chromosome inactivation (XCI) plays an important role in the phenotypic heterogeneity of X-linked disorders. However, the role of skewed XCI in XCI-escaping gene *SHOX* regulation is unclear. Here, we focused on a heterozygous deletion of *SHOX* gene enhancer with clinical heterogeneity. Using SNP array, we detected that the female proband with Leri-Weill dyschondrosteosis (LWD) carried an 857 kb deletion on Xp22.3 (encompassing *SHOX* enhancer) and a 5,707 kb large-fragment deletion on Xq25q26. XCI analysis revealed that the X-chromosome with the Xq25q26 large-fragment deletion was completely inactivated, which forced the complete activation of the other X-chromosome carrying *SHOX* enhancer deletion. While the Xp22.3 deletion locates on the escaping XCI region, under the combined action of skewed XCI and escaping XCI, transcription of *SHOX* gene was mainly from the activated X-chromosome with *SHOX* enhancer defect, involving in the formation of LWD phenotype. Interestingly, this *SHOX* enhancer deletion was inherited from her healthy mother, who also demonstrated completely skewed XCI. However, the X-chromosome with *SHOX* enhancer deletion was inactivated, and the normal X-chromosome was activated. Combing with escaping XCI, her phenotype was almost normal. In summary, this study was a rare report of *SHOX* gene enhancer deletion in a family with clinical heterogeneity due to skewed inactivation of different X-chromosomes, which can help in the genetic counseling and prenatal diagnosis of disorders in females with *SHOX* defect.

## Introduction

The short stature homeobox gene (*SHOX*), locating in the pseudoautosomal region (PAR1) of the short arm of the X and Y chromosomes, is one of the major growth genes in humans. In 1997, the *SHOX* gene was linked with the occurrence of short stature in Turner syndrome for the first time (Rao et al., 1997). Subsequently, *SHOX* haploinsufficiency has been demonstrated in individuals exhibiting different phenotypes, ranging from idiopathic short stature (ISS) to Lėri-Weill dyschondrosteosis (LWD) (Fukami et al., 2016). In LWD, the classic clinical features observed are short stature and Madelung deformity, which are also characterized by abnormal alignment of the radius, ulna, and carpal bones of the wrist (Seki et al., 2014). Additionally, a loss of both copies of *SHOX* results in the occurrence of Langer mesomelic dysplasia (LMD), which is a more severe disorder (Shears et al., 2002; Zinn et al., 2002).

The genetic defects underlying *SHOX* haploinsufficiency include copy-number variations (CNVs) and mutations, which not only occur in the coding region, but also in the regulatory elements of the *SHOX* gene (Binder, 2011). Among the regulatory elements, highly conserved non-coding DNA elements (CNEs) located several hundred kilobases downstream of *SHOX*, they have previously been identified as enhancers (Sabherwal et al., 2007). Numerous studies have shown that the *SHOX* gene enhancer plays an important role in the regulation of this gene to achieve optimal transcriptional efficiency. Furthermore, certain defects in the coding region along with deletions in the *SHOX* gene enhancer can also cause ISS or LWD, but the combined proportion in which both need to occur has not been well defined, since highly variable phenotypes are observed even if the same mutations run with in a family (Binder et al., 2004). Thus, a detailed study to determine the cause of these phenotypic differences was required.

X-chromosome inactivation (XCI) plays an important role in the phenotypic heterogeneity of X-linked disorders in females (Plenge et al., 2002; Renault et al., 2011; Sankaran et al., 2015; Torres and Puig, 2017; Juchniewicz et al., 2018). Due to skewed XCI, the same mutation in X-linked genes may result in the different phenotypes (Orstavik, 2009). While it was reported that skewed XCI might affect the phenotypes of patients with Xp22.3 complex rearrangement (Suzuki et al., 2016). However, the role of skewed XCI in the XCI-escaping gene *SHOX* was unclear. In this study, we have evaluated a case in which deletion of the *SHOX* enhancer was observed among members of a family with clinical heterogeneity due to differently skewed XCI. In this family, the proband with heterozygous deletion of the *SHOX* enhancer was a patient with LWD, although her mother harboring the same deletion was found to be almost healthy.

## Case Presentation

**Figure 1:**
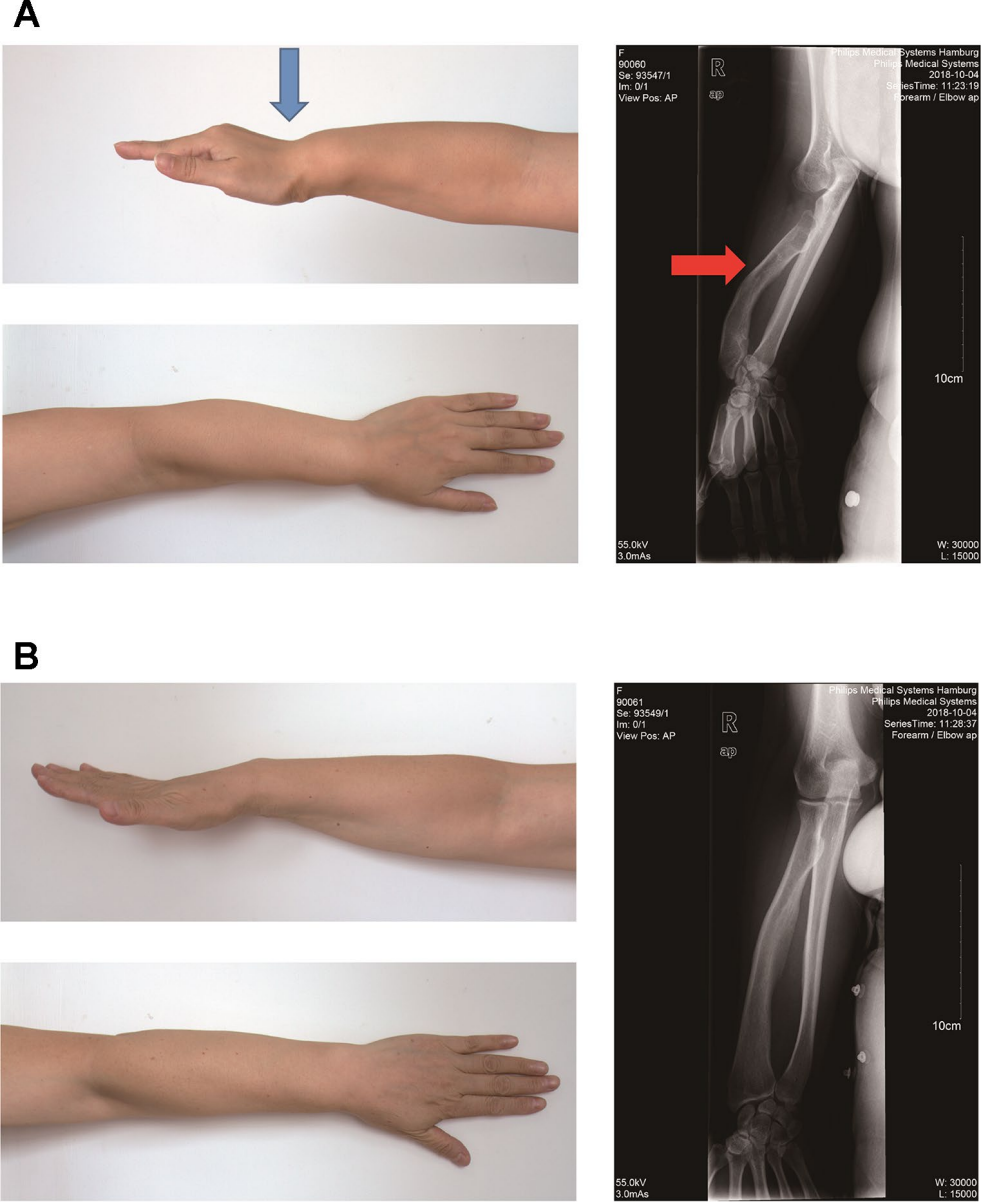
Photograph of the forearm of the proband II2 and mother I2. **(A)**. Photograph of the forearm of the proband II2 showing a Madelung deformity and bowing of the radius (blue and red arrows). This photograph shows the curve and shortening of the forearm, hand and wrist, the structure of which appears like that of a dinner fork. **(B)**. Photograph of the forearm of the mother I2 showing a normal phenotype.

The proband II2 is a 35-year-old Chinese woman with a short stature (150 cm, −1.1SD), bilateral Madelung deformity, bowing of the radius, and mesomelia affecting the arms, in particular, which was a typical LWD phenotype (**Figure 1A**). However, her parents and other family members were found to be healthy. It was observed that the mother was healthy with a height of 163cm (+1.1SD) and completely normal arms (**Figure 1B**).

**Figure 2:**
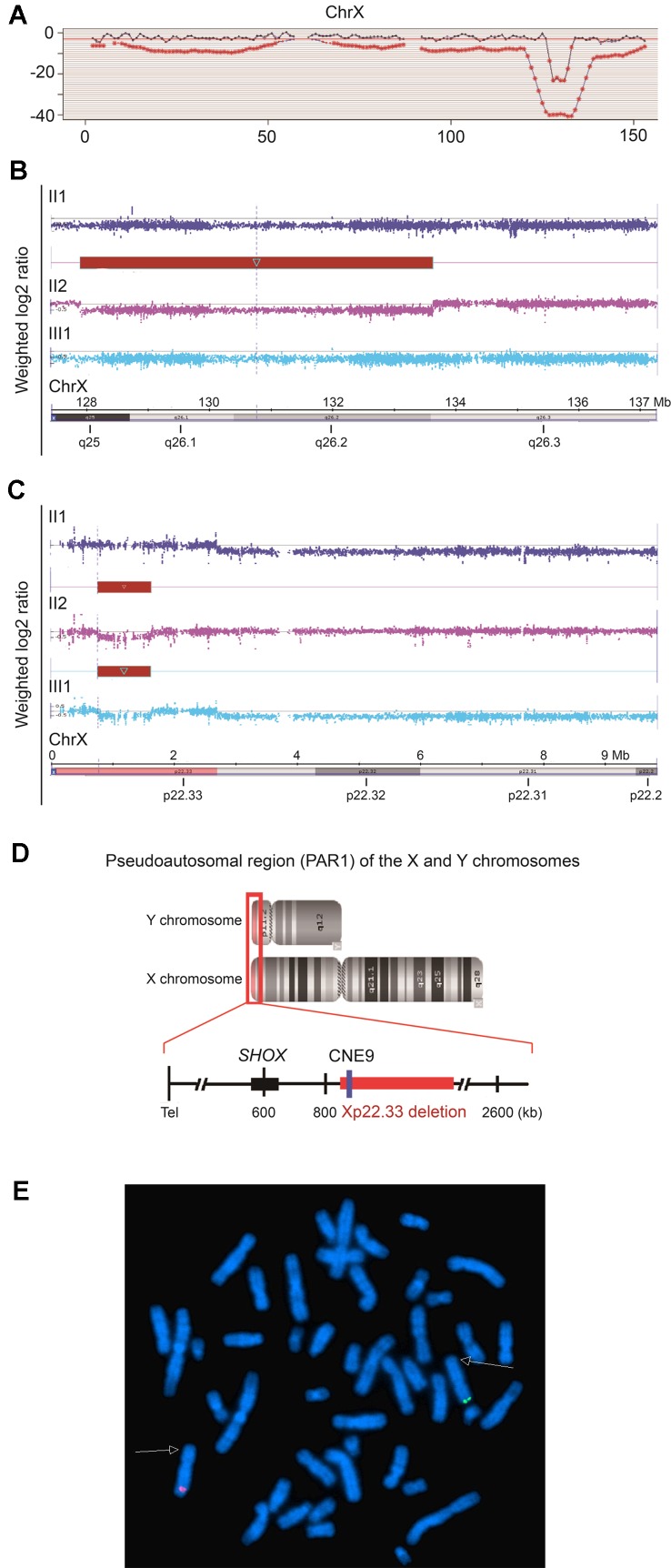
Characteristics of the 5,707 kb and 857 kb deletions of Xq25q26.3 and Xp22.33, respectively. **(A)**. Results of cfDNA screening of the pregnant proband. cfDNA screening study of the maternal plasma, illustrating an uncertain 6 Mb deletion in the long arm of the X-chromosome (128M–133M), Z score = −19.43. **(B** and **C)**. SNP array analysis of the fetus (III1) and the couples (II1, II2). **(B)**. The red bar indicates a heterozygous 5,707 kb deletion in Xq25q26.3 (chrX: 127,915,006–133,621,667) in the fetus (III1). **(C)**. The red bar indicates a heterozygous 857 kb deletion in Xp22.33 (chrX: 784,064–1,640,746) in the fetus (III1) and the pregnant proband (II2). **(D)**. Pseudoautosomal region (PAR1) of the X and Y chromosomes. The 857 kb deletion in Xp22.33 (red bar) was located 164 kb downstream of the *SHOX* gene (chrX: 585,079–620,146), including the evolutionarily conserved CNE9 (blue bar), which was the *SHOX* gene enhancer. **(E)**. For the proband II2, FISH experiment showed that the Xp22.3 deletion (detected by the probe RP11-1119O18, Spectrum Green) and the Xq25q26 deletion (detected by the probe RP11-313D19, Spectrum Red) located on the different X chromosomes, respectively.

The proband II2 arriving at our hospital was a pregnant female who was in her 14th week of gestation requesting for a non-invasive prenatal testing (NIPT). Triple tests on chromosomes 13, 18, and 21 showed a low risk in NIPT; however, a 6 Mb deletion in the long arm of the X-chromosome (Z-score was −19.43) was detected, which was anticipated to have a maternal origin (**Figure 2A**). In order to confirm the risk of this genetic disorder to the fetus, amniocentesis was subsequently conducted. Karyotyping and SNP array were performed and showed the presence of an 857 kb deletion in Xp22.33 (chrX: 784,064–1,640,746) in the male fetus III1. To verify the results, SNP array was also performed on samples of the pregnant woman II2 and her husband II1. Surprisingly, not only a deletion of 857 kb in Xp22.33, but also a 5,707 kb deletion (chrX: 127,915,006–133,621,667) in Xq25q26.3 was observed in the woman, the location of which was consistent with the result of the fetal NIPT (**Figures 2B, C** and **Supplementary Table 2**). SNP array results have been deposited in Gene Expression Omnibus (GEO), the accession number is GSE138489, as appended below: https://www.ncbi.nlm.nih.gov/geo/query/acc.cgi?acc=GSE138489. To explore the reason underlying the phenotypic differences between the proband II2 with LWD and her healthy mother I2, we also performed a PCR-based *HUMARA* assay to assess XCI patterns.

Finally, the fetus III1 with Xp22.33 deletion was born, his length was 50cm (−0.2 SD), weight 3.3 kg (+0 SD). On the latest examination at 8 months, his developmental milestones were almost normal, and his length was 72 cm (+0.3 SD), weight 11.7 kg (+2.2 SD).

This study was carried out in accordance with the recommendations of Ethics Committee of Women’s Hospital, School of Medicine Zhejiang University, and an informed consent was acquired from all the participants of this study in accordance with the Declaration of Helsinki. The study protocol was approved by the Review Board of the Women’s Hospital, School of Medicine, Zhejiang University in China.

## Materials and Methods

Materials and methods were in **Supplementary Materials** and **Supplementary Table 1**.

## Results

In order to explore the possible causes of the disease, we analyzed the two deletions (857 kb and 5,707 kb).The 5,707 kb deletion in Xq25q26.3 included 29 Online Mendelian Inheritance in Man (OMIM) genes. Among these, 9 genes (bold) were found to be morbid and might be associated with many X-linked diseases (**Supplementary Table 2**) but not with skeletal development. Additionally, the 857 kb deletion in Xp22.33 included 11 OMIM genes in the pseudoautosomal region (PAR1) of the X and Y chromosomes (**Figures 2B–D** and **Supplementary Table 2**). Interestingly, the *SHOX* gene enhancer, which is closely associated with skeletal development, was also located in the deletion region of Xp22.33. Further analysis revealed that it was located 164 kb downstream of the *SHOX* gene (chrX: 585,079–620,146) and included the evolutionarily conserved non-coding DNA element 9 (CNE9), which was the *SHOX* gene enhancer. We speculated that the 857 kb deletion in Xp22.33 had contributed to the *SHOX* gene mutation resulting in the LWD phenotype in case of the proband.

**Figure 3:**
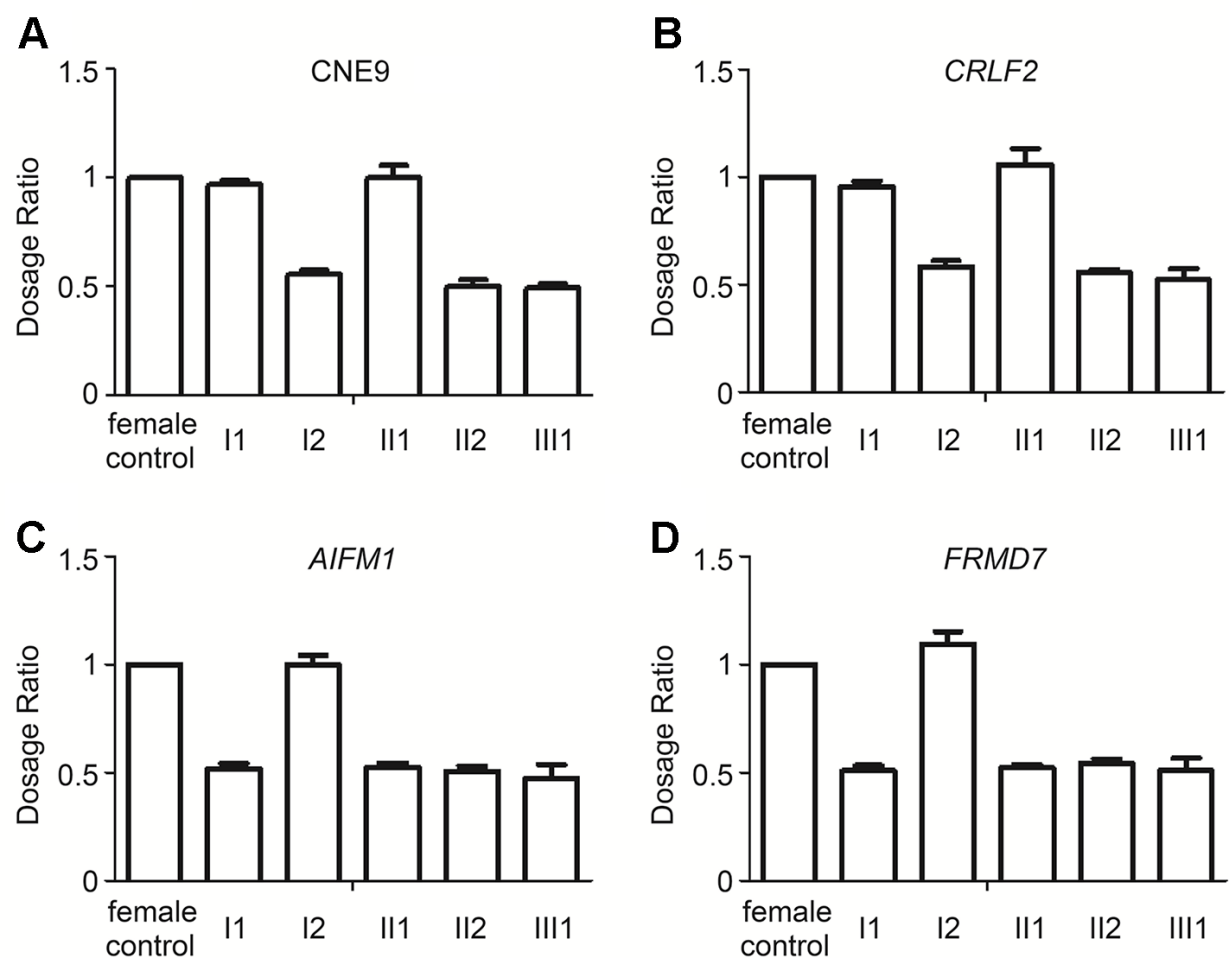
Relative ratio of the Xp22.33 (CNE9 and *CRLF2*) and Xq25q26.3 (*AIFM1* and *FRMD7*) regions in the family by qPCR. **(A** and **B)**. Xp22.33 region (CNE9 and *CRLF2*) was located in PAR1 of the X and Y chromosomes, including the *SHOX* enhancer. Dosages in normal female were equal, Xp22.33 region dosages in I1 and II1 were normal, and those in I2, II2, III1 with Xp22.33 deletion were half. **(C** and **D)** The *AIFM1* and *FRMD7* were present only in the X-chromosome. The *AIFM1* and *FRMD7* dosage in I2 were normal, while that in I1, II1, II2, III1 with Xq25q26.3 deletion was half. From the above data, it was elucidated that the Xp22.33 deletion in the proband II2 was inherited from her healthy mother I2, and the Xq25q26.3 deletion occurred *de novo*.

Although no other patients were exhibiting the LWD phenotype in her family, we detected the gene dosage of *CRLF2* and CNE9 (the *SHOX* gene enhancer in the deletion region of Xp22.33), *AIFM1* and *FRMD7* (deletion region of Xq25q26.3) of the female proband and her parents by qPCR (**Figure 3**). The Xp22.33 regions were both located in PAR1 of the X and Y chromosomes. Data obtained from a normal female was used as the control, and the dosages of CNE9 and *CRLF2* were normal in I1 and II1, and half in I2, II2, and III1 (**Figures 3A, B**). On the other hand, the Xq25q26.3 region existed only in the X chromosome. The gene dosages of *AIFM1* and *FRMD7* were normal in female I2, and half in I1, II1, II2, and III1 (**Figures 3C, D**). Above all, the Xp22.33 deletion in the proband II2 was inherited from her healthy mother I2, and the Xq25q26.3 deletion occurred *de novo*.

**Figure 4:**
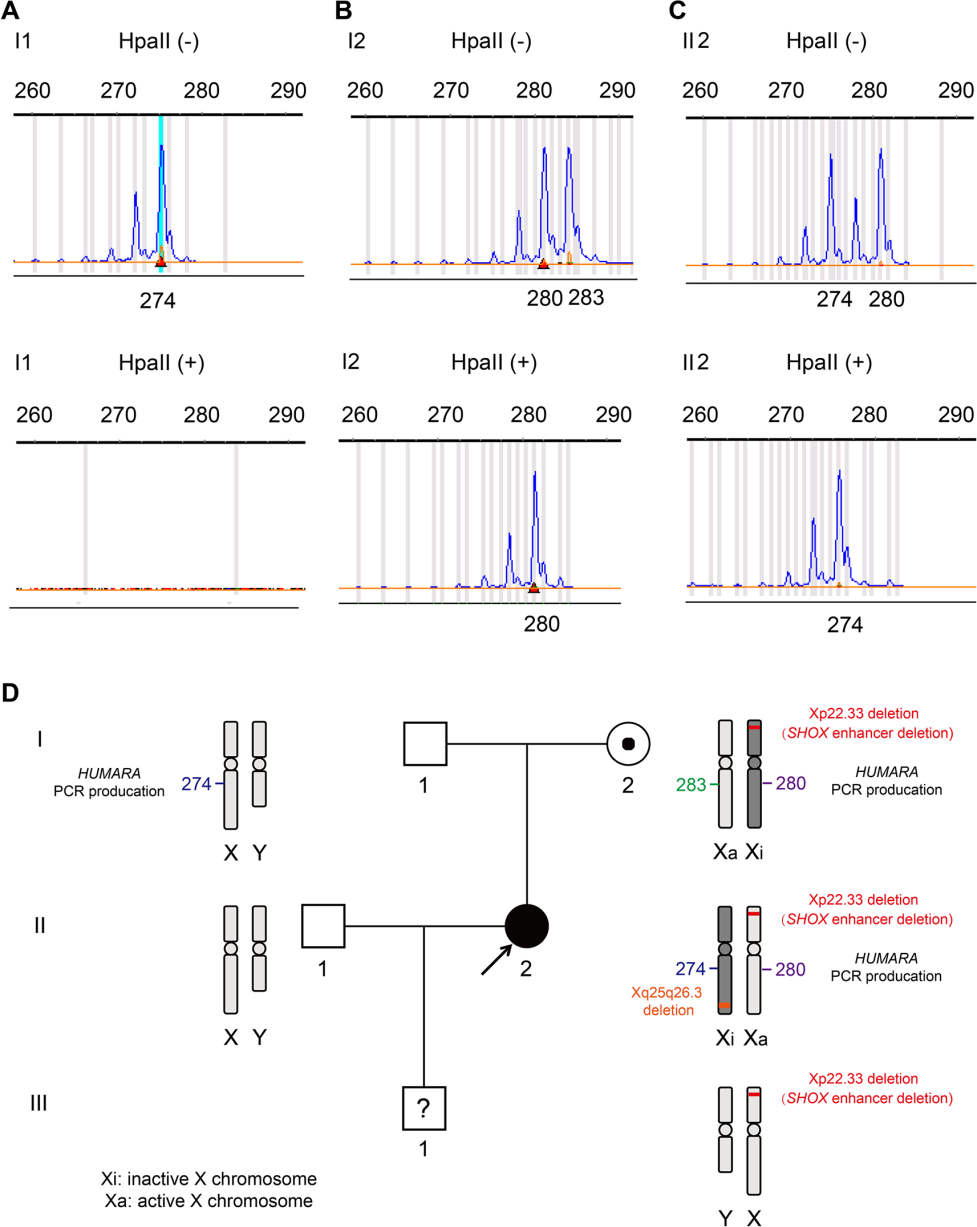
X-chromosome inactivation (XCI) pattern and linkage analyses were based on the polymorphic CAG repeat in exon 1 of the gene for androgen receptor (*HUMARA*). **(A)**. A peak of 274 bp for *HUMARA* was observed by assaying the undigested PCR product of I1 and no peaks were observed for the HpaII digested product. **(B)**. The undigested PCR product of I2 gave two peaks of 280 and 283 bp, each, while only one peak of 280 bp was observed with the HpaII digested product. I2 exhibited 100% skewing of XCI, and the inactivated X-chromosome was linked with the 280 bp peak of the *HUMARA* PCR products. **(C)**. The undigested PCR product of the proband II2 gave two peaks of 274 and 280 bp. One X-chromosome linked with the 280 bp peak of *HUMARA* was inhibited from the mother I2 and the other from the father I1. The product of HpaII digestion gave only one peak of 274 bp. II2 also demonstrated 100% skewing of XCI, but the inactivated X-chromosome was linked with the 274 bp peak of AR, which is different from that of the mother I2. **(D)**. Schematic diagram of Xp22.33 and Xq25q26.3 deletions, and *HUMARA* PCR products in the pedigree. It can be seen that Xp22.33 and Xq25q26.3 deletions in II2 are located on different X-chromosomes. Xp22.33 deletion (*SHOX* enhancer deletion) of I2 occurred in the X-chromosome, whose allele was linked with the 280 bp peak of *HUMARA* PCR. The X-chromosome was inactivated and delivered to the proband I2, but her X-chromosome was activated.

We further explored the reason underlying the phenotypic differences between the proband II2 with LWD and her healthy mother I2. As presented in **Figure 4**, a PCR-based *HUMARA* assay was performed to assess XCI patterns in II2 and I2. After digestion with the methylation-sensitive restriction enzyme HpaII, it was found that only the inactive X-chromosome could synthesize PCR products. The origin of the inactivated X-chromosome was determined by segregation analysis. The undigested PCR product of II2 gave two peaks of 274 bp and 280 bp, respectively. A single peak representing 274 bp was observed for the HpaII-digested product (**Figure 4B**), which indicated the inactivated allele inherited from her father I1, in whose samples one peak of 274 bp was assayed by PCR. The assay of the undigested PCR product of the mother I2 gave two peaks of 280 bp and 283 bp. A single peak of 280 bp was obtained by assaying the HpaII-digested product, which was different from that of the inactivated allele of II2. The above results showed that complete (100%) skewing of XCI was found in the proband II2 and her mother I2, which was different from that of the inactivated X-chromosome (**Figures 4C, D**). Segregation analysis revealed that the normal X-chromosome from her father I1 was completely inactivated (**Figure 4A**).

**Figure 4D** gives aschematic diagram of Xp22.33 and Xq25q26.3 deletions, and PCR product of *HUMARA* in the pedigree. Only Xp22.33 deletion (not Xq25q26.3 deletion) derived from II2 was seen in III1. Meanwhile, the FISH detection showed that the deletions of Xp22.33 and Xq25q26.3 in II2 were located on different X-chromosomes (**Figure 2E**). Both, Xp22.33 deletion (*SHOX* enhancer deletion) and 280 bp peak of *HUMARA* PCR products in II2, were derived from I2, which were linked to the same X-chromosome. However, the allele which gave a peak of 280 bp after *HUMARA *PCR was found to be activated in II2, but inactivated in I2 (**Figures 4B, C**).

## Discussion

Microdeletions in the region downstream of *SHOX* have been reported as the most common genetic defects in patients with LWD (Benito-Sanz et al., 2006; Marchini et al., 2016). Several evolutionarily conserved non-coding elements located downstream of *SHOX* (for example CNE4, CNE5, ECR1, and ECS4/CNE9), are known to act as enhancers (Benito-Sanz et al., 2005; Fukami et al., 2006; Huber et al., 2006; Rappold et al., 2007), and interactions between the *SHOX* gene and CNEs have been verified using *in vitro* and *in vivo* assays (Kenyon et al., 2011; Verdin et al., 2015). Moreover, the deletion of our case also included the limb enhancer with 563 basepair (bp) (chrX: 827,128–827,691), which had specific activity in the limb regions where SHOX functions, and it also contributed to the pathogenicity of deletions downstream of *SHOX* (Skuplik et al., 2018). In addition, microdeletions in the region that is further downstream of the previously known CNEs have recently been identified in patients with LWD features or short stature (Chen et al., 2009). Therefore, we concluded that the Xp22.33 deletion resulted in *SHOX* gene defect and contributed to the manifestation of abnormal skeletal phenotype in the proband. Interestingly, the proband was a patient with LWD, but her mother harboring the Xp22.33 deletion was almost normal.

Individuals with *SHOX* defect (for example CNE9 deletion) have a phenotype ranging from normal to LWD (Benito-Sanz et al., 2012; Bunyan et al., 2013; Marchini et al., 2016). Phenotypic differences have been explained by several hypotheses. For example, as a genetic modifier, *CYP26C1* variants can effect clinical manifestations of *SHOX* deficiency (Montalbano et al., 2016). For another example, it has described skewed X-inactivation patterns, which were caused by X-chromosomal rearrangements, can also effect phenotypes of Xp22.3 defect (Suzuki et al., 2016; Ogushi et al., 2019), which gives us an inspiration.

This further indicated the presence of differently skewed XCI in the proband and her mother, which suggested that clinical heterogeneity resulting from the deletion of the *SHOX* gene enhancer was caused by the skewed XCI. While *SHOX* is also an XCI-escaping gene (Carrel and Willard, 2005; Blaschke and Rappold, 2006; Sun et al., 2017). Skewed XCI and escaping XCI are both involved in our case. Expressions of the XCI-escaping genes on two X chromosome were not always equal, XCI-escaping genes in active X chromosome (Xa) were frequently in the state of preferential expression. LAURA CARREL et al. has indicated that the XCI-escaping gene REP1 was also expressed from the inactive chromosome (Xi), but the level of expression relative to Xa was reduced, it showed that skewed XCI were also existed in XCI-escaping genes (Carrel and Willard, 1999). In addition, Nathalie Fieremans et al. has also indicated that escaping XCI was often partial and incomplete with a lower expression from the inactive X chromosome. Skewed XCI of the XCI-escaping genes *DDX3X* and *SMC1A* were revealed in intellectual disability female patients (Fieremans et al., 2016). So we speculated that the *SHOX* enhancer was similar, based on the skewed XCI and escaping XCI theory, we can explain the current case reasonably.

According to one-hit of skewed XCI, defect in a single X-chromosome results in its inactivation, in order to remedy the defect. A large proportion of female carriers of severe X-linked disorders are asymptomatic and have severely skewed XCI, probably because of selectively mediated favorable skewing, thus suggesting preferential X-inactivation against the chromosome that harbors the mutation (Orstavik, 2009), for example ATR-X syndrome (Gibbons et al., 1992), dyskeratosis congenital (Ferraris et al., 1997), X-linked agammaglobulinemia (Moschese et al., 2000), and severe combined immunodeficiency (Li et al., 1998). In our study, X-chromosomes with deletion of the *SHOX* gene enhancer were completely inactivated in the proband’s mother I2, and the activated chromosomes were normal. Meanwhile, due to XCI-escaping, we speculated that transcription of SHOX was mainly from the activated X chromosome, which was normal. So it was observed that I2 was almost healthy and did not exhibit severe LWD phenotype.

According to the two-hit model of skewed XCI, if both the X-chromosomes are defective, the mutated X-chromosome with a more harmful mutation will be completely inactivated to avoid its adverse reaction, forcing the other to become active (Plenge et al., 2002; Orstavik, 2009). The hypothesis of “female X-linked two-hit model” has been used to support studies on multiple disorders, including X-linked intellectual disability (Plenge et al., 2002), *MECP2* duplication (Fieremans et al., 2014), and the Wiskott-Aldrich syndrome (Daza-Cajigal et al., 2013).

In our study, a deletion of 857 kb (including deletion of the *SHOX* gene enhancer) was found on one of the X-chromosomes of the proband II2, and another 5,707 kb deletion on the other. The larger deletion was likely more harmful. Hence, the X-chromosome with the 5,707 kb deletion was totally inactivated, and the other one with the *SHOX* gene enhancer deletion (857 kb deletion) was completely activated. Meanwhile, due to locating on XCI-escaping region, transcription of *SHOX* was partially from the inactive chromosome, but it was mainly origin from the active X-chromosome with *SHOX* gene enhancer deletion. Combing effect of skewed XCI and escaping XCI, the proband II2 presented severe LWD phenotype.

No skewed XCI were observed in the male. Perhaps the fetus III1 was too young to present abnormal phenotypes. It was reported that, during childhood, there was probably no relevant additional loss of height in patients with *SHOX* defect (Binder and Rappold, 1993), while mesomelic disproportion of the skeleton with shortening of the extremities can be evident first until in school-aged children and increase with age in frequency and severity (Ross et al., 2001). Although the phenotype of III1 is now normal, it doesn’t mean that it would be always normal, and final phenotype should be observed for long time.

XCI values depend on the tissues, but a general concordance of XCI patterns was observed among tissues from the same person (Bittel et al., 2008). Recently, Wen-Bin He et al. indicated that XCI pattern of amniocytes can predict the risk of dystrophinopathy in fetal carriers of DMD mutations (He et al., 2019). Therefore, we speculated XCI analysis in amniocytes cells may also contribute to predicting the phenotype of fetus with SHOX defect.

In summary, here we described a rare case of XCI–escaping gene *SHOX* enhancer deletion in a family with obvious clinical heterogeneity due to skewing inactivation of different X-chromosomes. Furthermore, we underlined the key role of skewed XCI and escaping XCI in the phenotype of X-linked disorders in females. It can help in the genetic counseling and prenatal diagnosis of disorders in females with *SHOX* defect.

## Data Availability Statement

Publicly available datasets were analyzed in this study. This data can be found here: GSE138489 (https://www.ncbi.nlm.nih.gov/geo/query/acc.cgi?acc=GSE138489).

## Ethics Statement

The studies involving human participants were reviewed and approved by Review Board of the Women’s Hospital, School of Medicine, Zhejiang University in China. Written informed consent to participate in this study was provided by the participants’ legal guardian/next of kin. Written informed consent was obtained from the individual(s), and minor(s)’ legal guardian/next of kin, for the publication of any potentially identifiable images or data included in this article.

## Author Contributions

MD designed the study. NIPT was carried out by HL; YS, and LW contributed the qPCR detection. YL and YQ performed the karyotyping, FISH and SNP array. XCI analysis was performed by YS and MC. YS and MD wrote the draft manuscript. YZ provided the imagological examination. All co-authors provided feedback on the estimates and contributed to the subsequent versions of the manuscript. All authors read and approved the final version of the manuscript.

## Funding

This study was supported by the National Natural Science Foundation of China (Grant Nos.81801441 and 81300532), the Key Research and Development Program of the Zhejiang province (Grant No.2019C03025), the National Key Research and Development Program of China (Grant Nos.2016YFC1000703 and 2018YFC1002702).

## Conflict of Interest

The authors declare that the research was conducted in the absence of any commercial or financial relationships that could be construed as a potential conflict of interest.
