# Supplementary material for: Heterozygous Deletion of the SHOX Gene Enhancer in two Females With Clinical Heterogeneity Associating With Skewed XCI and Escaping XCI
Source: Front Genet. 2019 Nov 6;10:1086. doi: 10.3389/fgene.2019.01086 (PMC6852097; doi:10.3389/fgene.2019.01086)
Supplement: Supplementary file 1 [file DataSheet_1.docx]

**Supplementary Materials**

**Non-invasive prenatal testing (NIPT)**

Maternal peripheral blood was collected into ablood collection tube containing the dipotassiumsalt of ethylenediamine tetraacetic acid (EDTA-K2).All standard procedures such as the isolation of cell-free DNA, library construction and sequencing were performed according to NIPT methods, which have been described previously ([Jiang et al. 2012](#_ENREF_2); [Qian et al. 2018b](#_ENREF_4)).

**Karyotype determination and SNP array**

The karyotype of the amniotic fetal cells was determined by conventional karyotyping of at least 30 blood lymphocytes arrested at metaphase using colchicines. The SNP array was performed according to the manufacturer’s instructions using the CytoScan™ HD array (Affymetrix, USA). The data was analyzed by the Chromosome Analysis Suite (ChAS) software (Affymetrix, Santa Clara, CA) based on the GRCh37/hg19 assembly.

**DNA extraction and qPCR**

Genomic DNA samples of the peripheral blood and fetal amniotic fluid were extracted using the GentraPuregene Kit (Qiagen, Germany). A qPCR was carried out using the SYBR green PCR master mix (Takara, Japan) and the Applied Biosystems 7900HT system. The primers used to conduct this test are listed in Supplementary Table 1. Melting curve analyses confirmed that all the primers were specific for their respective transcript. The ΔΔCt method was used to determine the relative levels of DNA, and the fold change was determined based on the value of 2^-ΔΔCt^.

**XCI analysis**

To assess the patterns of X-chromosome inactivation in affected females, a PCR-based *HUMARA* assay was performed. HpaII digestion resulted in the cleavage of the non-methylated *HUMARA* template DNA. Due to variable numbers of CAGrepeats between different alleles in the *HUMARA* sequence, two peaks corresponding to the maternal and paternal X chromosomes, which could be identified and quantified, were obtained after conducting the PCR assay([Allen et al. 1992](#_ENREF_1)).Genomic DNA was digested using the restriction enzyme HpaII (New England BioLabs) according to the manufacturer’s instructions. PCR assays were performed using the GoldStar Best MasterMix (CWBIO, Beijing). The primer sequences used for the PCR were as follows: FAM-labeled forward primer: 5'-TCCAGAATCTGTTCCAGAGCGTGC-3'; and reverse primer: 5'-GCTGTGAAGGTTGCTGTTCCTCAT-3'.Amplified products were analyzed using an ABI PRISM 3100 genetic analyzer.

**Fluorescence in situ hybridization (FISH)**

Peripheral blood sample of the proband II2 was analyzed by FISH with the probe (RP11-1119O18, Spectrum Green) on Xp22.3 and the probe (RP11-313D19, Spectrum Red) on Xq25q26. The slide hybridization and washes were performed according to standard FISH protocols ([Qian et al. 2018a](#_ENREF_3)).

**References**

Allen RC, Zoghbi HY, Moseley AB, Rosenblatt HM, Belmont JW. 1992. Methylation of HpaII and HhaI sites near the polymorphic CAG repeat in the human androgen-receptor gene correlates with X chromosome inactivation. Am J Hum Genet 51(6): 1229-1239.

Jiang F, Ren J, Chen F, Zhou Y, Xie J, Dan S, Su Y, Xie J, Yin B, Su W et al. 2012. Noninvasive Fetal Trisomy (NIFTY) test: an advanced noninvasive prenatal diagnosis methodology for fetal autosomal and sex chromosomal aneuploidies. BMC Med Genomics 5: 57.

Qian YQ, Fu XY, Wang XQ, Luo YQ, Chen M, Yan K, Yang YM, Liu B, Wang LY, Huang YZ et al. 2018a. A feasible diagnostic approach for the translocation carrier from the indication of products of conception. Molecular cytogenetics 11: 12.

Qian YQ, Wang XQ, Chen M, Luo YQ, Yan K, Yang YM, Liu B, Wang LY, Huang YZ, Li HG et al. 2018b. Detection of fetal subchromosomal aberration with cell-free DNA screening led to diagnosis of parental translocation: Review of 11344 consecutive cases in a university hospital. Eur J Med Genet.
