# Supplementary material for: Heterozygous Deletion of the SHOX Gene Enhancer in two Females With Clinical Heterogeneity Associating With Skewed XCI and Escaping XCI
Source: Front Genet. 2019 Nov 6;10:1086. doi: 10.3389/fgene.2019.01086 (PMC6852097; doi:10.3389/fgene.2019.01086)
Supplement: Supplementary file 2 [file Table_1.docx]

Supplementary Table 1. qPCR primers

| Genes | Primer sequences |
| --- | --- |
| β-globin-qPCR- forward primer | ACACAACTGTGTTCACTAGC |
| β-globin-qPCR- reverse primer | CAACTTCATCCACGTTCACC |
| CNE9-qPCR- forward primer | CAGCCTCCCTTTACCCAG |
| CNE9-qPCR- reverse primer | ACGGATGACAGGCGGTGT |
| *CRLF2*-qPCR- forward primer | TTGGGAATGCTTGAGAAC |
| *CRLF2*-qPCR- reverse primer | CGTGGCAACGGAGTGAGA |
| *FRMD7*-qPCR- forward primer | CCTTTCTCAGACCCTAAC |
| *FRMD7*-qPCR- reverse primer | TTGAATCACTGCTCCACT |
| *AIFM1*-qPCR- forward primer | ATAGGAGGGATGGCTTGG |
| *AIFM1*-qPCR- reverse primer | GCATTAGTATTTCAGAGGC |
