# Supplementary material for: Heterozygous Deletion of the SHOX Gene Enhancer in two Females With Clinical Heterogeneity Associating With Skewed XCI and Escaping XCI
Source: Front Genet. 2019 Nov 6;10:1086. doi: 10.3389/fgene.2019.01086 (PMC6852097; doi:10.3389/fgene.2019.01086)
Supplement: Supplementary file 3 [file Table_2.docx]

Supplementary Table 2. SNP array analysis of the proband II2

| Type | Chr. | Region | Start | End | Length | OMIM genes |
| --- | --- | --- | --- | --- | --- | --- |
| Loss | X | Xq25q26.3 | 127,915,006 | 133,621,667 | 5,707 | *SMARCA1 (300012),* ***OCRL (300535,*** ***Dent disease 2, Lowe syndrome,XLR)****, APLN (300297),* ***XPNPEP2 (300145,*** ***Angioedema induced by ACE inhibitors, susceptibility to)****, SASH3 (300441),* ***ZDHHC9 (300646,*** ***Mental retardation, X-linked syndromic, Raymond type)****, UTP14A (300508), BCORL1 (300688), ELF4 (300775),* ***AIFM1 (300169,*** ***Combined oxidative phosphorylation deficiency 6,*** ***Cowchock syndrome,*** ***Deafness, X-linked 5, XLR)****, RAB33A (300333), SLC25A14 (300242), GPR119 (300513), ENOX2 (300282), ARHGAP36 (300937),* ***IGSF1 (300137,*** ***Hypothyroidism, central, and testicular enlargement, XLR)****, STK26 (300547),* ***FRMD7 (300628,*** ***Nystagmus)****, MBNL3 (300413), HS6ST2 (300545), USP26 (300309), TFDP3 (300772), GPC4 (300168),* ***GPC3 (300037,*** ***Simpson-Golabi-Behmel syndrome, type 1, XLR)****, MIR19B2 (300722), MIR20B (300950), MIR106A (300792),* ***PHF6 (300414,*** ***Borjeson-Forssman-Lehmann syndrome, XLR)****,* ***HPRT1 (308000,*** ***HPRT-related gout,*** ***Lesch-Nyhan syndrome, XLR)*** |
| Loss | X | Xp22.33 | 784,064 | 1,640,746 | 857 | *CRLF2 (400023), CRLF2 (300357),* ***CSF2RA (306250, ,* *Surfactant metabolism dysfunction, pulmonary, 4), CSF2RA (425000, ,* *Surfactant metabolism dysfunction, pulmonary, 4)****, IL3RA (308385), IL3RA (430000), SLC25A6 (300151), SLC25A6 (403000), ASMTL (400011), ASMTL (300162), P2RY8 (300525)* |
